# First Nation Peoples’ nutrition and exercise group programmes: transforming success through the lifeworld

**DOI:** 10.1080/17482631.2021.1990197

**Published:** 2021-11-09

**Authors:** Lisa Urquhart, Karin Fisher, Kerith Duncanson, Karen Roberts, Simon Munro, Clinton Gibbs, Leanne Brown

**Affiliations:** a Department of Rural Health, College of Health, Medicine and Wellbeing, The University of Newcastle, Coffs Harbour, New South Wales, Australia; b Department of Rural Health, College of Health, Medicine and Wellbeing, The University of Newcastle, Tamworth, New South Wales, Australia; c The Priority Research Centre for Digestive Health and Neurogastroenterology, Callaghan, New South Wales, Australia; d College of Health, Medicine and Wellbeing, The University of Newcastle, Callaghan, New South Wales, Australia; e Galambila Aboriginal Health Service, Coffs Harbour, New South Wales, Australia; f Mid North Coast Local Health District, Port Macquarie, New South Wales, Australia

**Keywords:** Indigenous people, Habermas, critical hermeneutics, literature interpretation, shared decision-making

## Abstract

**Purpose:**

Previous literature has applied system-focused structures to understand the success of First Nations Peoples’ nutrition and exercise group programmes. Existing system-focused measures have included biomedical outcomes, access and service utilization. By broadening the focus of programme success beyond the system, we can evaluate programmes from a First Nations Peoples’ lifeworld perspective. Critical hermeneutics and yarning using a lens of Habermas’ Theory of Communicative Action to the literature has the potential to transform understandings of “success” in First Nations Peoples’ nutrition and exercise group programmes.

**Methods:**

In this literature interpretation, we explored the critical success factors from a lifeworld perspective, giving scope to go beyond a system perspective to include a cultural, social or personal perspective.

**Results:**

Our yarning led us to understand that there is a communicative relationship between explicit system structures and implicit lifeworld concepts that are critical success factors for First Nations nutrition and exercise group programmes. We have developed a set of reflective questions to guide others in considering a lifeworld perspective.

**Conclusions:**

Our findings represent a shift away from success measured by the dominant power structure to respect the lifeworld culture, knowledges and values of First Nations Peoples towards shared understanding and mutual decision-making.

## Introduction

The damaging impact of colonization on health and well-being continues to negatively and unequally affect First Nations Peoples living in the developed and well-resourced countries of Australia, Canada, Aotearoa New Zealand and the USA (Pulver et al., 2010). First Nation’s health disparities are complex, rooted in racism and exacerbated by the inequitable distribution of social determinants of health (Eckermann et al., 2010; Harris et al., 2012). Westernized systems contribute to health disparities through the suppression and devaluation of traditional protective health, physical activity and food practices informed by First Nations ways of knowing, being and doing (Eckermann et al., 2010).

Previous literature reviews focused on First Nations Peoples’ nutrition and exercise group programmes have explicitly applied system-focused structures as measures of success. These reviews evaluated success through biomedical outcomes, access, service utilization and models of service (see examples: Gwynn et al., 2019; Pressick et al., 2016; Schembri et al., 2016). Gwynn et al. (2019) extended their review by applying existing ethics frameworks and guidelines to the articles in their review (see National Health and Medical Research Council, 2010, 2018). Through this process, they examined evidence of Australian Aboriginal and Torres Strait Islander community research initiation, governance, engagement and capacity building within the existing literature. While their review extended the evaluation of the literature by considering cultural components, the success of the programmes was still determined through a system lens, as opposed to understanding success through a cultural, social or personal lens.

The Ngaa-bi-nya framework developed by Williams (2018) offers an alternative evaluation of critical success factors for culturally considered research. The framework expands understandings of critical success factors beyond a system-focus to consider a “lifeworld” perspective. It prompts researchers and programme designers to consider cultural, social and personal elements as critical success factors that are guided by social norms, knowledges, values, ethics and ways of care giving (Williams, 2018). By broadening the focus of the success of a programme beyond the system-based measures, programmes and research are able to be designed, implemented and evaluated from a First Nations Peoples’ lifeworld perspective (Straits et al., 2012; Williams, 2018).

Programmes that are underpinned by First Nations People’s lifeworld perspectives and culturally considered evaluation methods offer a way to decolonize health and well-being programmes. Lifeworld perspectives are complex and diverse but may include intergenerational connections (Mcintosh et al., 2021), traditional ways of sharing knowledge, language and connection to country (Williams, 2018). Culturally considered programme evaluations that celebrate First Nations People's ways of knowing, being and doing may contribute to decolonization by interrogating critical success factors from a lifeworld perspective (Smith, 2012).

The interpretive nature of hermeneutics offers a way to uncover implicit meanings, and critical theory can expose, interrogate and challenge conventional social structures by working against the oppression of disempowered minority groups (Crotty, 1998; Trede et al., 2008). To understand how success represents First Nations Peoples’ values and worldviews requires an unmasking of critical success factors beyond system requirements. The aim of this literature interpretation is to apply critical hermeneutics through a lens of Habermas’s Theory of Communicative Action (Habermas, 1984, 1987) to transform understandings of success in First Nations Peoples’ nutrition and exercise group programmes through a lifeworld perspective.

## Methodology

### Interpretive process

Our literature interpretation was informed by critical hermeneutics, which allowed us to interrogate and critically address the impact of power and domination on the understanding of meaning and its representation in a text (Kögler, 2012). We interlinked yarning with critical hermeneutics in an effort to decolonize the Western paradigm. Yarning is a method of research and communication style, which is rigorous, credible and valued by Australian Aboriginal people (Bessarab & Ng’andu, 2010; Zwar et al., 2017). Through active listening, mutual respect and layering knowledge, yarning is an Aboriginal method of research that is consensus-building, meaning making and innovative (Yunkaporta, 2019).

**Figure 1.**
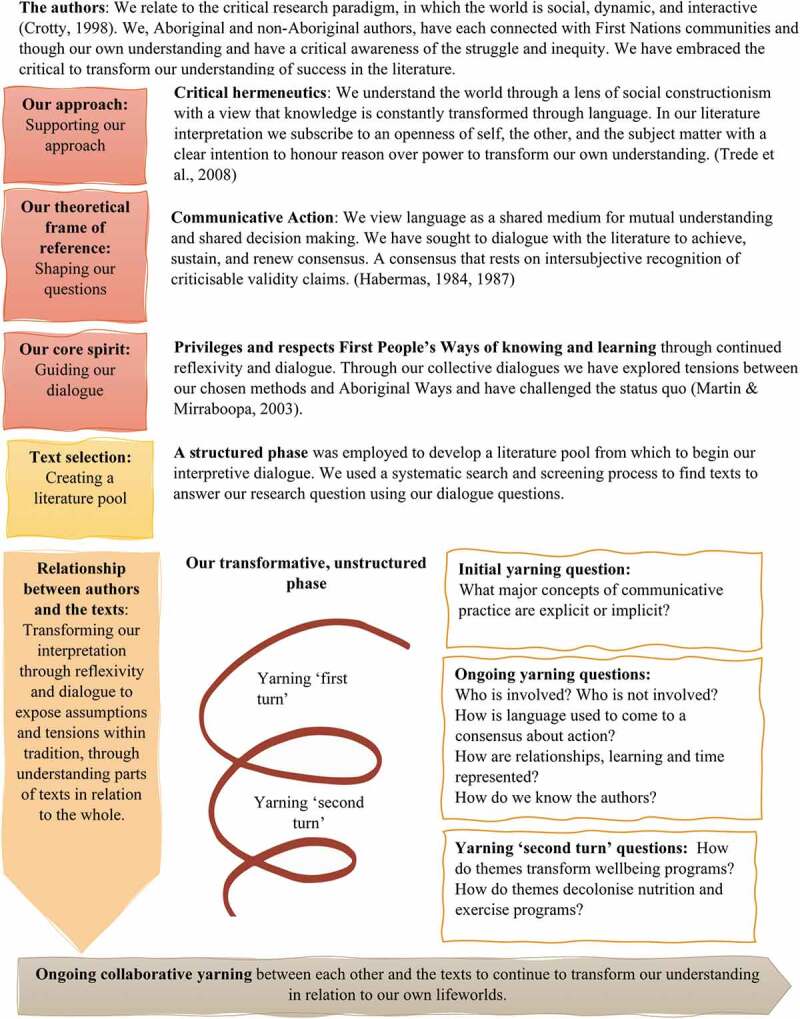
Frame of reference and methods which developed through our review process and informed the literature interpretation to transform understandings (figure inspired by Hudson & Croker, 2018)

We suggested a set of *question and answers* for our yarning to critically reflect on the status quo and power in our chosen texts and *the hermeneutic circle towards transformation* for iterative returns to the texts to transform our interpretation beyond our horizon of understanding (see Figure 1) (Hudson & Croker, 2018; Trede et al., 2008). We believed this approach would guide us towards transformative understandings by focusing our awareness on power, authority and dominance in language and communication (Trede et al., 2008). We wanted to use this literature interpretation to move beyond “a transaction of information” and the constraints of our own unconscious biases, by intentionally exposing and interrogating the interests and reasoning of research (Trede et al., 2008). Central to this approach is the possibility of this newly gained knowledge disrupting the dominant research dialogue towards emancipation (Trede et al., 2008).

### Theoretical frame of reference

Our process was underpinned by Habermas’ Theory of Communicative Action through which interpretation, understanding and consensus towards emancipatory action are the primary concern (Figure 1) (Habermas, 1984, 1987). Habermas describes two types of social actions that are either strategic (the system) or communicative or (the lifeworld). He describes that strategic action drives system success through efficiency, money and control (Habermas, 1984, 1987). Conversely, Habermas describes the lifeworld to embody our culture, traditions, social relations and inner self. Through the critical medium of language, our lifeworld is reproduced through the intersubjective understanding and mutual decision-making of participants involved in a communicative action (Habermas, 1984, 1987).

Whilst both “worlds” need to exist, Habermas warns that when the system encroaches on the lifeworld, the lifeworld becomes controlled by the system (Kemmis, 2001). Alternatively, if communicative action guides decision-making and actions, the lifeworld becomes liberated from the system. A liberated lifeworld celebrates critical success factors of our cultural, social and personal lifeworlds.

### Identity and terminology—embracing our lifeworlds

We are a group of Australian Aboriginal (KR, SM and CG), and Australian non-Aboriginal (LU, KF, KD and LB) authors who are working together (Figure 1). We embrace the diverse identities, cultures and connections to language, social and nation groups informing each individual’s lifeworld (Urquhart et al., 2020).

KR identifies as a proud Dunghutti woman with family ties to Birpai, Gumbaynggirr and Bundjalung Nations. She has over 20 years of experience working in Aboriginal community health settings. Her strong leadership and community connections as an Aboriginal health worker have enabled interlinkages between Aboriginal and Western knowledge sharing methods to ensure ongoing Aboriginal leadership, governance, and relevance of research to the Aboriginal community.

SM is an Aboriginal Research Academic; his Anaiwan and Kamilaroi Nation heritage strongly informs his research. His cultural, educational and community knowledge brings a strong decolonizing influence on his work in research and higher education.

CG is an Aboriginal Health Promotion Officer and a proud Muruwari man. He has a Master's in Public Health and over 14 years of experience in planning, implementing, and evaluating Aboriginal nutrition and exercise group programmes.

LU is a PhD student and experienced dietitian. KR invited LU to work closely with her and the community. Through her engagement over the past 4 years, LU has built strong relationships and collaborative dialogues with community members, stakeholders and KR.

KF, KD and LB are LU’s research supervisors who have developed a collaborative working relationship with KR, CG and SM, including as part of an existing research project (Urquhart et al., 2020).

We have selected terminology (Supplement 1) to respectfully refer to First Nations people in this article. We recognize that specific terminology applies to a given context and that there are tensions around the meaning of terms used to describe colonized groups who seek to reclaim their identities and define themselves through their social realities (Simeone, 2020). We acknowledge that the terminology used in this article does not encompass the rich and varied First Nations identities or lifeworlds.

## Methods

### Stages of interpretation

There were two stages to this literature interpretation: an initial structured phase and a subsequent unstructured phase (see Figure 1). The structured phase incorporated the development of a structured search strategy, article selection and initial charting of article characteristics. The unstructured phase integrated two yarning turns of the literature and an ongoing cultural mentoring arrangement between KR and LU.

### Structured phase

The literature pool that informed our yarning consisted of original research articles and grey literature, initially sourced from a structured search strategy (Supplement 2) developed with assistance from a consultant librarian at the University of Newcastle, Australia (Arksey & O’Malley, 2005; Greenhalgh et al., 2018). The search was completed in November 2018 and updated in November 2020 by LU.

Electronic databases Medline, CINAHL, Scopus, Embase and Informit were searched. Included articles detailed First Nations Peoples’ nutrition and exercise programmes in group face-to-face community settings, for people over 18 years, in Australia, Canada, Aotearoa New Zealand and USA. There was no timeframe limit, as we felt that all previous literature had relevance to the research question. Literature reviews and articles not in English were excluded.

### Article selection

All articles identified by the database searches were collated into EndNote (X9, Clarivate Analytics, Philadelphia, USA) where duplicates were identified and removed. The remaining articles were uploaded to Covidence (Veritas Health Innovation, Melbourne, Australia), which was used as a tool to manage the review process. Two reviewers independently reviewed the title and abstract of each article and considered relevance to inclusion criteria. Reviewers retrieved full texts if either reviewer thought that an article was potentially eligible for inclusion (LU, KF, KD and LB). Two reviewers independently assessed full texts to determine whether they met the inclusion criteria. Reviewers noted conflicts and discussed their differences. If reviewers could not agree, a third reviewer determined whether the article met the inclusion criteria.

LU entered initial data from the literature pool into Excel (Version 2103, Microsoft Corporation, Redmond, USA) to record: authors, publication year, study population, aims of study, description of group programme, facilitators and setting. This process enabled the authors to grasp an initial understanding of the breadth of the literature pool.

## Unstructured Phase

### Yarning “first turn”

Parallel to this initial charting, LU, KR, KF, KD and LB began yarning (first turn) with the literature pool. Thirty-eight texts from the literature pool were included and represented positivistic, interpretive and critical perspectives (Crotty, 1998).

We used a *question and answer* approach to critique representations of success in the literature pool (Figure 1). LU used NVivo (Version 12.4, QSR International Pty Ltd) to code text sections that answered the questions, as well as new information that did not “fit” with our pre-existing questions. Alongside our yarning, LU maintained a reflexive diary, where she documented her reframed and transformed understandings.

LU, KR, KF, KD and LB held meetings every 2–4 weeks over 6 months to yarn about emerging themes, tensions and perspectives. At each meeting, LU made diary notes about the group yarning and interpretations. Each reviewer dialogued with a sub-sample of the literature pool prior to the next collaborative yarning. Throughout this process, reviewers were acutely aware of the role of power, authority and dominance and shared and discussed their clear intention to honour reason over power (Trede et al., 2008).

During the yarning process, LU engaged in ongoing yarning with Aboriginal Elder and mentor KR. They worked together to transform initial interpretations and themes from the literature pool. As theme development matured, LU, KR, KF, KD and LB discussed concerns that there was not adequate First Nation’s voice represented in the process. What we viewed as our “unsettled understandings”, could have been disguised as our own “extended” interpretations. To challenge our thinking, we invited further perspectives from alternative lifeworlds. We approached SM and CG, with whom we had previous research relationships, to join as co-researchers and yarning partners. SM and CG agreed to collaborate as part of a yarning “second turn”.

### A yarning “second turn”

KR, CG, SM and LU joined in a yarning “second turn”. This involved five meetings over 4 months to draw out, unsettle and transform initial interpretations. Yarning partners mutually decided the agenda, time and frequency of meetings. Meetings were conducted and recorded via Zoom (Zoom Video Communications, San Jose, California). LU audio-visually recorded each meeting and made diary notes. Prior to each meeting, LU listened back to the recording to draw out themes. Each meeting involved a yarning between each other, the literature pool, emerging themes, and our own lifeworld understandings. Co-authors also yarned with additional reference texts (see Eades, 2013; Helin, 2013), which were not part of the initial literature pool. These texts resonated with us and probed us to critique our interpretations surrounding lifeworld concepts.

**Figure 3.**
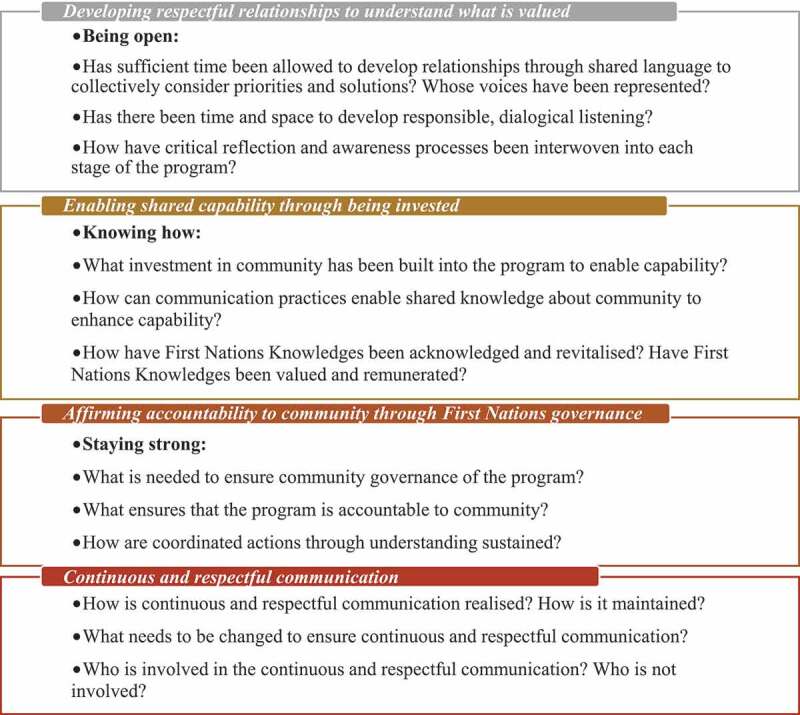
Reflective questions to guide engagement and research considering a lifeworld perspective

This literature interpretation did not use all texts from the literature pool in the same way, which may appear as a methodological limitation or oversight. However, our evolving interpretation fit our critical hermeneutic intention, to transform understanding through hermeneutic turns from alternative, socially constructed perspectives to interrogate power and strategic action. We view this yarning process as a continuous process, inclusive of many voices towards emancipation. The findings detailed in this article are the results of our interpretations of the literature, which we transformed through the progression of our two yarning turns (see Figure 1). The findings represent mutual decisions of the overall authorship group’s transformed understandings. From our yarning, we cultivated a set of reflective questions (Reflections, Figure 3). These questions were developed as a group and are informed by our own experiences and transformed understandings.

## Findings

**Figure 2.**
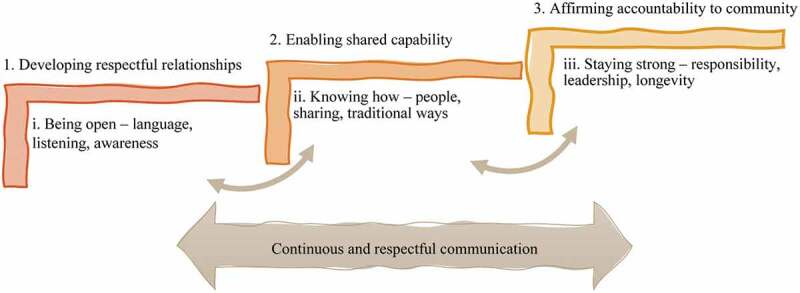
Three interdependent communicative relationships between system structures (1,2,3) and lifeworld concepts (i, ii, iii) supported by continuous and respectful communication

Our yarning with each other and the literature about First Nations Peoples’ nutrition and exercise group programmes revealed an interdependent relationship between an explicit system structure and implicit lifeworld concept, presented as vertical steps in Figure 2. Success in First Nations nutrition and exercise group programmes was represented as three interdependent communicative relationships involving system structures and lifeworld concepts. Continuous and respectful communication underpins the three interdependent system structures and lifeworld concepts. Based on the findings presented below, we discuss each system and lifeworld relationship and focus on the interplay between the system structures and lifeworld concepts. Each system/lifeworld notion is presented as discrete interdependent units.

### Developing respectful relationships to understand what is valued

Communicative action is interpreted as a relationship between the system construct of developing respectful relationships to understand what is valued and the lifeworld concept of “being open” (Figure 2). The system construct comprises the establishment of relationships that are meaningful, formal or informal and includes shared voice. Respectful relationships must begin to develop prior to any programme planning or decision-making to understand “what is valued” by community. The formation of respectful relationships in initial programme development offers an opportunity to understand each other, power-share and maintain or build community governance from different viewpoints (Oetzel et al., 2020; Prodan-Bhalla et al., 2017; Tipene-Leach et al., 2013). Respectful relationships determine a culture of open communication, and collective solutions through community leadership, capability, skills, and trust within the programme governance (Oetzel et al., 2020). The quote below demonstrates this structur:e

The first task of the [project] was to build a working relationship with the team that created “oneness” that would extend to the program participants. Walking a path of equality is essential to creating a new path for actual relationships to occur. (Prodan-Bhalla et al., 2017, Seven Sisters Healthy Heart Project, Canada)

Formal or explicit respectful relationships established a shared voice for mutual understanding between community and external parties. The genuine establishment of formal programme mechanisms ensured meaningful relationships were communicatively orientated towards mutual understanding (Curtis et al., 2004; Jernigan, 2010; Oetzel et al., 2020; Rosas et al., 2020; Rowley et al., 2000; Vallesi et al., 2018). In these circumstances, there was an opportunity for each individual’s voice to inform the programme, and together “what is valued” by the community was jointly explored towards a mutual understanding and programme success. A representative example of respectful relationships valid to the community is illustrated here:

the partnership between Aboriginal Elders and Aboriginal Health Workers, a partnership which gives the Team its legitimacy within the local Aboriginal community. (Curtis et al., 2004. Aunty Jean’s Good Health Team, Australia)

In the same vein, respectful relationships represented an opportunity for “what is valued” to be heard and recognized:

Priorities and concerns of each partner were presented at council meetings, and solutions were considered collectively. (Castro et al., 2009, Full Circle Diabetes Program, USA)

Respectful relationships created an inclusiveness between individuals, which provided validity to the broader community. It was apparent through our yarning that “what is valued” varied between individuals involved in a programme and there was different representation of “what is valued” between programmes in the literature pool. Our second yarning turn provided depth and perspective to understanding the cultural context of “what is valued” through the lifeworld concept of “being open”, discussed below.

#### Being open

Underpinning a successful programme is the lifeworld concept of “being open”. Understanding “what is valued” through “being open” is represented through the qualities of shared language, dialogical listening, and critical awareness for each member involved in the communicative process. Members of a communicative process included participants, programme facilitators, researchers or health care workers.

The quality of shared language, which was inclusive, intersubjective and co-created established a dialogue that was both communicative and decolonizing, as described in this quote:

The language used in the Program outline, symbolises accessibility, inclusion, being active, and having fun. (Curtis et al., 2004, Aunty Jean’s Good Health Team, Australia)

Shared language that aimed to understand “what is valued” required a common dialect, including shared gesture and speech, such as traditional languages, words or phrases. The meaning and use of traditional words or phrases was shared by community members, who also helped others such as researchers to understand the significance to the community (Curtis et al., 2004; Janssen & Nelson, 2014; Sinclair et al., 2020; Tipene-Leach et al., 2013; Warbrick et al., 2020).

Drawings, photographs, and metaphors were important to many communities as ways to convey meaning and feelings, as well as to add cultural relationality to a dialogue about a programme (Curtis et al., 2004; Janssen & Nelson, 2014; Stefanich et al., 2005). Throughout the literature pool, meaningful visuals linked traditional ways of sharing knowledge, exemplified by:

Shared story telling between staff and participants, involving imagery and analogies to convey information about medical concepts and participants’ experiences. (Dimer et al., 2013, Heart Health – for our people, by our people, Australia)

Our second yarning turn raised concerns regarding the use of strategic tokenistic imagery or symbols that were perceived as superficial or reinforcing stereotypes (Willging et al., 2006). It also fostered further considerations around seeking permission and remunerating for the use of meaningful lifeworld words and images in programmes and related research.

Another quality, dialogical listening strengthened understanding of “what is valued” via shared relational and situational conversations. In the literature pool, these types of conversations enhanced learning about community values, particularly in the planning and evaluation phases of a programme (Curtis et al., 2004; Parmenter et al., 2020; Tipene-Leach et al., 2013; Wicklum et al., 2019; Willging et al., 2006). The use of active listening, part of dialogical listening, was represented in the literature pool through reciprocal respect and trust:

Participation in the talking circle also forced me to be present and to LISTEN. (Prodan-Bhalla et al., 2017, Seven Sisters Healthy Heart Project, Canada)

Rather than trying to merge individual voices into a single “strong” voice, we yarned, in our first and second turns, about listening to all voices. We recognized “polyphonic listening” (refer to reference text Helin, 2013) as hearing what is not said, said quietly, or only once. For designers and facilitators of a diabetes prevention programme for urban American Indian women, “polyphonic listening” meant respectfully listening to both older and younger voices when redesigning a culturally relevant programme (Willging et al., 2006).

“Polyphonic listening” also encompasses listening to one’s own inner voice within the interplay of other’s voices. Our second turn considered how a listener moves away from the tradition of Western monologues that have dominated First Nations Peoples’ health programmes, to incorporate other voices as well as one’s own, as demonstrated below.

it was understood that the [team] had to listen to the community and learn how to use these cultural values as strengths of the program. (Bachar et al., 2006, Cherokee Choices, USA)

Our yarning approached listening as an embodied activity involving other senses in the act of listening, to become a way of “seeing” through a multisensory way of being (see reference text Helin, 2013 for further detail). Our second yarning turn saw “seeing” as relevant to First Nation’s lifeworld entities such as spirituality (Prodan-Bhalla et al., 2017; Struthers et al., 2003; Wicklum et al., 2019). A quote that illustrates a connection between spirituality and listening is as follows:

“You need your spirituality or connection. You need to get to your centre, so you know there’s a place to go, up and down,” and “if we let people know what we mean [about diabetes] … what we’re saying from the heart, they will listen.” (Struthers et al., 2003, Talking Circles, USA)

Dialogical listening supports the idea of responsible decolonizing engagement in the transaction of speech and listening in a moment. This can create a sense of “we-ness” through relationality and continuous, respectful communication toward a shared learning about “what is valued” in the lifeworld.

The third quality, critical awareness, was necessary for each person involved in the dialogue. Critical awareness stimulated individuals to understand their own lifeworld’s agenda, values and motivations to begin to understand “what is valued” by other people. Individuals who were aware of their own motivations were able to consciously act on these, make them known to others, and have others able to agree or disagree on “what is valued” in a programme. An example of critical awareness is shown belo:w

… we came to understand that we approached our work from profoundly different bases of knowledge and experience, which in turn led us to different ways of practicing. (Prodan-Bhalla et al., 2017, Seven Sisters Healthy Heart Project, Canada)

Through both yarning turns, we acknowledged that self-reflection and awareness were a journey that took time for each individual. For people who were not from a particular community, time was needed to understand what was valued by community, establish relationships and become aware of the self (Castro et al., 2009; Jernigan, 2010; Langwell et al., 2014; Mendenhall et al., 2010). This process required intentional dialogue, critical reflection and actions that were accountable to the lifeworld. This concept of critical awareness is exemplified in the below example:

I had relocated to the area and held the status of outsider, or at best “insider-outsider” as a Native American … To reach out to the community I knew I would need the guidance and mentoring from an elder community member … (Jernigan, 2010, The Santa Clara Valley Diabetes Community Action Project, USA)

Self-awareness and critical reflection extended beyond what was said to include gesture and non-verbal body language (Janssen & Nelson, 2014; Jernigan, 2010; Prodan-Bhalla et al., 2017). Through critical awareness, non-verbal cues may become more recognizable to people from different cultures (discussed in the reference text by Eades (2013)). For example, Eades (2013) describes those periods of silence, which may be uncomfortable for some, as a positively valued part of many conversations for Australian Aboriginal people. The silence of listening and reflection from each member in a communicative action demonstrated “being open” to another’s lifeworld.

Critical awareness about the programme location, time and actions, such as sharing a meal or snack, were important aspects towards understanding “what is valued” by individuals (Abbott et al., 2012; Fredericks et al., 2005; Harris & Curtis, 2005; Thompson et al., 2000). The below example illustrates how critically reflecting on the logistics of a programme can help to learn about “what is valued”.

[the programme] had been offered during the evening at dinnertime, no meal had been provided to the participants … . Transportation had also not been provided. I felt the problem … had been in its introduction to the community and in its delivery. (Jernigan, 2010, The Santa Clara Valley Diabetes Community Action Project, USA)

The lifeworld qualities of critical awareness, along with shared language and dialogical listening through “being open” support facilitation of respectful relationships towards understanding “what is valued” for programme success. This interdependent relationship can then foster investment from the community and stakeholders in a programme, through “knowing how” to enable shared capability.

### Enabling shared capability through being invested

Through both yarning turns, we identified that enabling shared capability was a critical success factor that depended on having the means and resources available to invest in knowledge, advocacy, and employment underpinned by communication practices necessary to enable a shared capability. Shared capability was recognized as enhanced confidence, capacity and ability in the context of well-being programmes. The system construct of shared capability through investing one’s (or community) time, hope and effort into a programme related communicatively to the lifeworld concept of “knowing how” (see Figure 2).

We understood that investment in a programme is related to its perceived value at both the individual and community levels. The term “invested” also indicated to us that there is capital (capacity) to invest, including assets like knowledge, skills and health. Investment occurred through recognizing, honouring and, where required, co-building capability, using continuous and respectful communication. This quote from the literature pool illustrates investment from the local community:

Our guiding principles were developed principally by members of the research and intervention team who were part of the local community, and had an inherent understanding and in-depth knowledge of the local cultural values and community networks (Tipene-Leach et al., 2013, Ngāti and Healthy Prevent Diabetes Project, Aotearoa New Zealand)

Meetings can be a place to develop shared capabilities through communication practices, the following quote extends this idea:

Meetings with tribal agencies and community groups facilitated planning and capacity building, which led to development of the community action plan. (Bachar et al., 2006, Cherokee Choices, USA)

Through our yarning with each other and the literature pool, we understood shared capability enabling to be a two-way approach that worked to enable capability of community and others, such as non-community member researchers (Mau et al., 2010), health workers or health students (Harris & Curtis, 2005). Enabling shared capability needs was defined by the community, and worked to strengthen health knowledge (Curtis et al., 2004; Firth et al., 2012; Payne, 2013), cultural knowledge (Keith et al., 2017), community resources (Firth et al., 2012) and skills (Castro et al., 2009; Leonard et al., 1987). It also built cultural humility, flexibility and understanding of community customs for non-community members (Davey et al., 2014; Langwell et al., 2014; Ziabakhsh et al., 2016).

The following quote is an example of communication practices to enable capability as a two-way process:

Providers and researchers learned about American Indian culture, belief systems, and manners–all because they were allowed into the American Indian community itself. In turn, community members gained more insights into and regarding how Western medicine is oriented, and thereby secured better understanding(s) into providers’ habitudes and perspectives in care delivery. (Mendenhall et al., 2010, The Family Education Diabetes Series, USA)

Programmes fostered the opportunity to include the broader community in a programme by enabling capability through shared investment (Davey et al., 2014; Jernigan, 2010; Oetzel et al., 2020; Payne, 2013; Rowley et al., 2000; Warbrick et al., 2020). The following quote demonstrates how enabling capability became a catalyst to advocate for health within a community:

As they gained skills and had positive experiences planning and executing these activities, participants developed the confidence necessary to become advocates for self-management. (Castro et al., 2009, The Full Circle Diabetes Program, USA)

Investing in enabling shared capability created an internal momentum within the community by employing community members (Braun, 2015; Jernigan, 2010; Rowley et al., 2000). This action flowed on to community members supporting peers towards mutual action through understanding (Castro et al., 2009; Curtis et al., 2004; Mendenhall et al., 2010; Parmenter et al., 2020).

In summary, enabling shared capability is a two-way approach, supported by continuous and respectful communication. Investing in knowledge, advocacy, and employment enhanced confidence for programme and community members. Through our second yarning turn, we understood the relationship between the lifeworld concept of “knowing how” to support this system structure as a critical success factor for programmes.

#### Knowing how

Inherent throughout enabling shared capability was the lifeworld concept of “knowing how” through qualities of invested key people, non-linear knowledge sharing and revitalizing First Nations Peoples’ knowledges.

Invested key people were the advocates of a programme, navigating between the lifeworld and the system. Through our yarning, we understood these people to include First Nations Health Workers, Elders and community members who had “one foot” in each “world”. Key people were familiar and trusted by the community and maintained continuous and open communication, represented below.

The Program has been built around the community’s capacity to work together for better health outcomes, with the Elders leading the way. (Curtis et al., 2004, Aunty Jean’s Good Health Team, Australia)

Invested key people developed and maintained strong connections and trust with community through repeatedly investing their time and “showing up” (Firth et al., 2012; Jernigan, 2010; Parmenter et al., 2020; Sinclair et al., 2020; Warbrick et al., 2020). They had a relational connection beyond the programme through family and friends, and reciprocally through these relationships, community members were invested in the programme (Bachar, 2006 ; Fredericks et al., 2005; Jernigan, 2010; Thompson et al., 2000). The quote below demonstrates how a programme became part of the lifeworld.

The women joined for health reasons, but soon learned that walking time is also family time; time to sort out everyday problems; time to talk and laugh. (The Heart Foundation, 2009, Yuendumu Heart Foundation Walking Group, Australia)

Key people, with strong community relationships navigated the development of relationships with people who were not from communities such as health professionals or researchers (Firth et al., 2012; Fredericks et al., 2005; Parmenter et al., 2020; Wicklum et al., 2019). Those not from community needed to invest time and maintain continuous and respectful communication to develop relationships with invested key people (Jernigan, 2010; Parmenter et al., 2020).

Our second yarning turn revealed a tension for key people as they navigated between the lifeworld of “knowing how” and system of “enabling shared capability”. The tension surrounded system rigidities when advocating for lifeworld flexibilities. Key people displayed resilience and solidarity when navigating system rigidities around policy, timing, funding and governance (Firth et al., 2012; Jernigan, 2010; Langwell et al., 2014; Tipene-Leach et al., 2013; Ziabakhsh et al., 2016).

Non-linear knowledge sharing is the second quality related to investment in enabling shared capability. Through our second yarning turn, we established that learning and knowledge were intertwined. Invested learners involved in a nutrition and exercise programme defined their own learning needs in relation to their lifeworld knowledge and experiences. Through a communicative process, learners were the knowledge holders of their own lifeworld of food and movement (Parmenter et al., 2020; Warbrick et al., 2020; Ziabakhsh et al., 2016).

Knowledge sharing, towards enabling mutual capability, was flexible and multidirectional, where praxis was valued over didactic information (Prodan-Bhalla et al., 2017; Vallesi et al., 2018). Multidirectional learning responded to lifeworld needs such as sorry business (a time of mourning in Australian Aboriginal communities) (Firth et al., 2012) and family responsibilities (Parmenter et al., 2020), as outlined in this quote:

if you miss out in one lecture or you come in half-way through, you just go back and start again. So it’s just a continuous circle, you know? And that’s how things are with Aboriginal education. Everything’s in circles, not in lines. (Vallesi et al., 2018, Heart Health – for our people, by our people, Australia)

Praxis-based knowledge sharing was valued through shared activities including the use of artefacts like weaving (Doyle et al., 2016; Walters et al., 2012), imagery (Jernigan, 2010) or story (Dimer et al., 2013; Firth et al., 2012; Jernigan, 2010). Other shared activities that connected to culture, including fishing (Harris & Curtis, 2005), hunting (Heart Firth et al., 2012; Foundation, 2009; Rowley et al., 2000) or dance (Kaholokula et al., 2017), were also valued as relaxed, unconstrained opportunities to enable shared capability. Cooking or a communal meal also supported knowledge sharing through investing time and effort and, enabling capability in a relaxed and positive atmosphere (Curtis et al., 2004; Doyle et al., 2016; Firth et al., 2012; Mendenhall et al., 2010).

The following quote illustrates how praxis-based knowledge sharing methods enable capability:

Social interaction, chats, walks, and meal times all provided opportunities for participants to share their stories, and to increase their awareness of strategies of self-management and to provide opportunities for these to be put into practice. (Harris & Curtis, 2005, Self Management Camp, Australia)

The third quality, revitalizing First Nations Peoples’ knowledges, was identified as protective of health and well-being, and connected to identity and self-worth (Sinclair et al., 2020; Wicklum et al., 2019). This concept required those involved, including facilitators, researchers and health care workers to respect, recognize and remunerate cultural knowledge holders of languages, customs, and expertise, to enable a shared capability and understanding (Brown et al., 2020; Coppell et al., 2009; Curtis et al., 2004; Harris & Curtis, 2005; Tipene-Leach et al., 2013; Warbrick et al., 2020).

The below quote demonstrates how revitalized lifeworld knowledge informed a programme:

Community members have detailed knowledge of their local environment, organisations and networks. (Coppell et al., 2009, Ngāti and Healthy Prevent Diabetes Project, Aotearoa New Zealand)

It was not clear through our yarn with the literature pool as to whether and, to what extent, programmes remunerated cultural knowledges or the extent to which remuneration was valued by First Nations knowledge holders.

### Affirming accountability to community through First Nations governance

The third system structure from our literature interpretation is affirming accountability to the community through First Nations governance. We understood that affirming accountability to the community occurred through establishing First Nations governance, supported by continuous and respectful communication. This structure is related to the lifeworld concept of “staying strong” to build a third critical success factor (Figure 2).

Affirming accountability to the community was represented through explicitly developing equitable partnerships (Tipene-Leach et al., 2013), mapping the transfer of ownership (Mobbs et al., 2003), and the transformation of a committee into a community governance team (Curtis et al., 2004; Mendenhall et al., 2010; Thompson et al., 2000). Through our second yarning turn, we recognized that affirming accountability to the community through First Nations governance was represented as a journey from within the community, stemming from community voices (Firth et al., 2012; Fredericks et al., 2005) to benefit community members (Brown et al., 2020).

Throughout our yarns, we acknowledged and respected the responsibilities of providing governance for a nutrition and exercise programme. The meaning and value of a programme for the community determined First Nations governance rather than an obligation for the community to run an already established programme. From the initiation of the programme, accountability to the community via continuous and respectful communication worked to maintain First Nation’s governance. We understood that First Nation’s governance has a relationship with the lifeworld concept “staying strong” towards a successful programme.

#### Staying strong

Supporting governance was the lifeworld concept of “staying strong” through qualities of ongoing responsibility, reshaping and reforming through community leadership, and long-lasting programmes.

The quality of ongoing responsibility ensured accountability to the lifeworld. Those involved in a programme were accountable through continuous communication with the community, from the initial design, through to the evaluation and dissemination of programme results (Curtis et al., 2004; Mendenhall et al., 2010; Oetzel et al., 2020). The following quote demonstrates an ongoing responsibility to the lifeworld through community governance.

Data collection was carried out collaboratively, as we recognized that American Indian community members would be more trusting of these processes if other American Indians and their families were involved (Mendenhall et al., 2010, The Family Education Diabetes Series, USA).

Our second yarning turn highlighted a tension of responsibility between lifeworld collaboration and system funding (Mendenhall et al., 2010; Thompson et al., 2000). For example, Tipene-Leach et al. (2013) describe a tension of responsibility between securing system funding through a large grant proposal and the importance of developing collaborative lifeworld governance within the community.

Another lifeworld quality was reshaping and reforming a programme through community governance. This quality was demonstrated through inviting the broader community to become involved (Firth et al., 2012; Harris & Curtis, 2005; Rowley et al., 2000), forming support groups (Jernigan, 2010; Mendenhall et al., 2012; Payne, 2013) or community changing the structure and content of the programme and evaluation focus (Parmenter et al., 2020; Rosas et al., 2020; Wicklum et al., 2019; Ziabakhsh et al., 2016). The following quote illustrates an example of flexibility through community governance:

Waminda continues to respond to community need, by ensuring a constant, cyclical approach, community consultation and ongoing involvement which shapes the program (Firth et al., 2012, Waminda’s Wellbeing Program, Australia)

Programme longevity was the third lifeworld quality related to affirming accountability to the community through First Nations governance. This quality extended a programme beyond brief research interventions towards sustainable actions, with longevity through communication, trust, and support from the community (Firth et al., 2012; Rowley et al., 2000; Wicklum et al., 2019). Through valued community support, a programme transcended the whims of system pressures, such as political elections, and short-term research contracts (Bourgette-Henry et al., 2019; Oetzel et al., 2020; Thompson et al., 2000). Below is an example that exemplifies long-lasting coordinated programme actions despite system challenges.

While the Program still faces existing and emerging challenges, it has been established on a firm foundation of local leadership and community strength. (Curtis et al., 2004, Aunty Jean’s Good Health Team, Australia)

## Reflections

We have represented three interdependent relationships between explicit system structures and implicit lifeworld concepts. The interplay of these relationships characterizes critical programme success factors that require continuous and respectful communication. Our interpretations have made explicit the implicit lifeworld concepts that contribute to the success of First Nations’ nutrition and exercise group programmes.

Lifeworld concepts are the often-implicit critical success factors for First Nations’ nutrition and exercise group programmes. Lifeworld concepts extend beyond Westernized system-focused structures as measures of success. First Nations Peoples’ ways of knowing, being and doing (Martin & Mirraboopa, 2003) underpin lifeworld concepts through cultural considerations including traditional ways of sharing knowledge, language and connection to country (Smith, 2012; Williams, 2018). By fostering and celebrating lifeworld concepts, along with system structures programmes can work towards decolonizing First Nations People’s health and well-being.

Through our yarning in this review process, we have developed a set of reflective questions that explore lifeworld perspectives. These questions were developed through our yarning as a group and are underpinned by our own experiences and transformed understandings. The questions aim to prompt communities, individuals, researchers and health professionals to pursue mutual understanding and shared decision-making when planning, designing, implementing and evaluating a First Nation’s nutrition and exercise group programme (see Figure 3). By adopting reflective questions, those involved can continue to transform and decolonize the narrative of First Nations health communicatively towards a successful programme.

Our reflective questions build on other tools such as Harfield et al. (2020)’s *Aboriginal and Torres Strait Islander Quality Appraisal Tool* and Huria et al. (2019)’s *CONSIDER statement* to provide opportunities for programmes, research approaches and resultant publications to explicitly describe approaches of quality health research from a First Nations lifeworld perspective. Pragmatically, our reflective questions encourage authors to clarify how their understanding, decision-making, and actions developed when reporting on programmes. As an example, through documenting the two-way learning of participants and facilitators cooking together, exchanging favourite recipes and sharing a meal, the relationship between the programme system and lifeworld becomes explicit. In this instance, the critical success factor of enabling shared capability through being invested is supported by the lifeworld concepts of praxis-based non-linear knowledge sharing and invested key people.

While this review data and findings are limited to adult First Nation People’s Nutrition and Exercise group programmes. It is possible that these relationships, and reflective questions, may apply to, draw out and underpin critical programme success factors in other health programme contexts. These relationships may also be expanded to other contexts. For example, Mcintosh et al. (2021) explored intergenerational connection as a critical success factor to enable shared capability in well-being between grandparents and grandchildren in Aotearoa-New Zealand. Hence, intergenerational connections may be an expanded lifeworld concept in that particular context.

## Conclusion

This literature interpretation was co-authored by Australian Aboriginal and Australian non-Aboriginal collaborators and used yarning and a critical hermeneutic approach through a lens of communicative action to transform our understandings. Our yarning has led us to understand that there is a communicative relationship between explicit system structures and implicit lifeworld concepts that are critical success factors for First Nations nutrition and exercise group programmes. These relationships are contextually-dependent and underpinned by continuous and respectful communication. Our findings represent a shift from success measured by the dominant power structure towards shared understanding and mutual decision-making that embraces the lifeworld culture, knowledges and values of First Nations Peoples.

## Supplementary Material

Supplemental Material

## Data Availability

The data that support the findings of this study are available from the corresponding author, LU, upon reasonable request.
