# Supplementary material for: First Nation Peoples’ nutrition and exercise group programmes: transforming success through the lifeworld
Source: Int J Qual Stud Health Well-being. 2021 Nov 9;16(1):1990197. doi: 10.1080/17482631.2021.1990197 (PMC8583907; doi:10.1080/17482631.2021.1990197)
Supplement: Supplemental Material [file ZQHW_A_1990197_SM2323.zip › Supplementray/Supplement_1_Terminology.docx]

**Supplementary Box 1:** **Selected Terminology used in our literature interpretation**

**References:**

**First Nations**: The authors have used this term to respectfully refer to people who identify as First Peoples from nations, lands and territories around the world, and wish to acknowledge their diverse and rich cultures.

**Australian Aboriginal**: The authors have used this term to respectfully refer to First Peoples from the mainland of Australia.

**Torres Strait Islander**: The authors have used this term to respectfully refer to First Peoples who are from the Torres Strait Islands of Australia.

**Maori:** The authors have used this term to respectfully refer to First Peoples who are from the Islands of Aotearoa New Zealand.

**Indigenous Canadian:** The authors have used this term to respectfully refer to First Peoples who are from Canada.

**American Indian:** The authors have used this term to respectfully refer to First Peoples who are from the USA.

**Alaskan Native:** The authors have used this term to respectfully refer to First Peoples who are from Alaska, USA.

**Native Hawaiian:** The authors have used this term to respectfully refer to First Peoples who are from the Hawaiian Islands, USA.

**Sources**: National Museum of the American Indian (2021), Simeone (2020), Australian Institute of Aboriginal and Torres Strait Islander Studies (2018), Eckermann et al. (2010), Keith et al. (2017), Taonui (2017)

Australian Institute of Aboriginal and Torres Strait Islander Studies. 2018. *Indigenous Australians: Aboriginal and Torres Strait Islander people* [Online]. Canberra, Australia. Available: <https://aiatsis.gov.au/explore/articles/indigenous-australians-aboriginal-and-torres-strait-islander-people> [Accessed 20 May 2020].

Eckermann, A.-K., Dowd, T., Chong, E., Nixon, L., Gray, R. & Johnson, S. 2010. People Centred Care. *In:* Eckermann, A.-K., Dowd, T., Chong, E., Nixon, L., Gray, R. & Johnson, S. (eds.) *Binan Goonj.*

Keith, J. F., Stastny, S. N., Agnew, W., Brunt, A. & Aune, P. 2017. Life skills at a tribal college: A culturally relevant educational intervention. *Journal of Extension,* 55.

National Museum of the American Indian. 2021. *The impact of words and tips for using the right terminology - Am I using the right word?* [Online]. Smithsonian. Available: <https://americanindian.si.edu/nk360/pdf/Impact-of-Words-Tips-for-Using-Appropriate-Terminology.pdf> [Accessed 18 March 2021].

Simeone, T. 2020. *Indigenous Peoples: Terminology Guide* [Online]. HillNotes: Research and Analysis from Canada's Library of Parliment. Available: <https://hillnotes.ca/2020/05/20/indigenous-peoples-terminology-guide/> [Accessed 18 March 2021].

Taonui, R. 2017. *Ngā tuakiri hōu – new Māori identities* [Online]. Available: <http://www.TeAra.govt.nz/en/nga-tuakiri-hou-new-maori-identities/print> [Accessed 18 March 2021].
