# Supplementary material for: First Nation Peoples’ nutrition and exercise group programmes: transforming success through the lifeworld
Source: Int J Qual Stud Health Well-being. 2021 Nov 9;16(1):1990197. doi: 10.1080/17482631.2021.1990197 (PMC8583907; doi:10.1080/17482631.2021.1990197)
Supplement: Supplemental Material [file ZQHW_A_1990197_SM2323.zip › Supplementray/Supplement_2_Search strategy.docx]

| Population | ‘Aboriginal’ OR ‘Indigenous’ OR ‘First Nation’ OR ‘Indian’ OR ‘Maori’ OR ‘Inuit’ OR ‘Eskimo’ OR ‘Sami’ OR ‘Metis’ OR ‘Pima’ OR ‘Australian Aboriginal’ OR ‘Torres Strait Islander’ OR ‘Native’ OR ‘Oceanic Ancestry Group’ OR ‘Koori’ OR ‘Goori’ OR ‘Tiwi’ OR ‘American native continental ancestry group’ |
| --- | --- |
| Intervention | (‘Nutrition’ OR ‘diet’ OR ‘cooking’ OR ‘food intake’ OR ‘exercise’ OR ‘physical activity’ OR ‘fitness’ OR ‘lifestyle’ OR ‘wellness’ OR ‘wellbeing’ OR ‘health education’ OR ‘lifestyle’ or ‘holistic’ or ‘self-management’) OR  ((‘community’ or ‘peer’ or ‘elder’ or ‘health worker’ or ‘culture’) within three words of (‘based’ or ‘led’ or ‘group’ or ‘support’ or ‘manage’ or ‘develop’ or ‘implement’ or ‘design’ or ‘run’ or ‘evaluated’ or ‘ran’ or ‘program’ or ‘circle’ or ‘activity’ or ‘intervention’ or ‘course’ or ‘project’ or ‘session’ or ‘directed’)) OR ((‘aboriginal’ or ‘indigenous’ or ‘first people’ or ‘first nation’) within three words of (‘ways of knowing’ or ‘ways of being’ or ‘ways of doing’ or ‘ways of learning’)) OR ((‘aboriginal’ or ‘indigenous’ or ‘first people’ or ‘first nation’) within three words of (‘ontology’ or ‘epistemology’ or ‘pedagogy’ or ‘axiology’)). |
| Comparator | Not applicable |
| Outcome | (‘diabetes’ OR ‘hyperglycaemia’ OR ‘glucose intolerance’ OR ‘metabolic syndrome’ OR ‘Obesity’ OR ‘hypertension’ OR ‘cardiovascular disease’ OR ‘weight loss’ OR ‘kidney disease’ OR ‘chronic disease’ OR ‘high cholesterol’) |

**Supplementary 2: Systematic search strategy**

**Table 1: PICO search strategy**

**Table 2: Medline search strategy**

| 1. exp american native continental ancestry group/ or oceanic ancestry group/ |  |
| --- | --- |
| 2. ((aborigin* or indigenous or native*) adj5 (australi* or canad* or new zealand or americ*)).mp. [mp=title, abstract, original title, name of substance word, subject heading word, floating sub-heading word, keyword heading word, protocol supplementary concept word, rare disease supplementary concept word, unique identifier, synonyms] |  |
| 3. Torres Strait* Islander*.mp. |  |
| 4. (maori* or sami* or inuit* or eskimo*).mp. or Inuits/ |  |
| 5. (first nation* or first people*).mp. |  |
| 6. (metis or pima* or koori* or goori* or tiwi*).mp. [mp=title, abstract, original title, name of substance word, subject heading word, floating sub-heading word, keyword heading word, protocol supplementary concept word, rare disease supplementary concept word, unique identifier, synonyms] |  |
| 7. 1 or 2 or 3 or 4 or 5 or 6 |  |
| 8. Diabetes Mellitus, Type 2/ or diabet*.mp. |  |
| 9. Metabolic Syndrome/ or Hyperglycemia/ or high blood glucose.mp. |  |
| 10. impaired glucose tolerance.mp. or Glucose Intolerance/ |  |
| 11. OBESITY/ or OBESITY, MORBID/ or OBESITY, ABDOMINAL/ or obes*.mp. |  |
| 12. cardiovascular diseases/ or exp hypertension/ or heart diseas*.mp. |  |
| 13. OVERWEIGHT/ or weight-loss.mp. or weightloss.mp. or weight loss.mp. |  |
| 14. kidney disease.mp. or Kidney Diseases/ or renal disease*.mp. |  |
| 15. chronic disease*.mp. or Chronic Disease/ or cardiometabolic.mp. or cardi* metabol*.mp. |  |
| 16. Hypercholesterolemia/ or Atherosclerosis/ or high cholesterol.mp. or Arteriosclerosis/ |  |
| 17. Coronary Disease/ or Dyslipidemias/ or dyslipidaemia.mp. or Hyperlipidemias/ |  |
| 18. 8 or 9 or 10 or 11 or 12 or 13 or 14 or 15 or 16 or 17 |  |
| 19. Health Education/ or Diet/ or Health Promotion/ or (nutrition education or nutrition* intervention).mp. or Feeding Behavior/ |  |
| 20. (nutrition* or healthy eating or diet* or food* or food intake*).mp. |  |
| 21. exp Healthy Diet/ or exp Life Style/ |  |
| 22. Cooking/ or cook*.mp. or healthy eating*.mp. |  |
| 23. EXERCISE/ or exercise.mp. |  |
| 24. (physical activit* or physical inactivit* or fit* or sedentary).mp. |  |
| 25. (walk* or gym* or train*).mp. or WALKING/ |  |
| 26. (wellness or well-ness or well ness or health* or wellbeing or well-being or lifestyle or life-style or life style or holistic or self manag* or self-manag*).mp. |  |
| 27. 19 or 20 or 21 or 22 or 23 or 24 or 25 or 26 |  |
| 28. ((communit* or peer* or elder* or health work* or cultur*) adj3 (base* or led* or group* or support* or manag* or develop* or implement* or design* or run* or eval* or ran* or program* or circle or activit* or intervent* or course* or project* or session* or direct*)).mp. [mp=title, abstract, original title, name of substance word, subject heading word, floating sub-heading word, keyword heading word, protocol supplementary concept word, rare disease supplementary concept word, unique identifier, synonyms] |  |
| 29. ((aborigin* or indigenous or first people* or first nation*) adj3 (ways of knowing or ways of being or ways of doing or ways of learning)).mp. |  |
| 30. ((aborigin* or indigenous or first nation* or first people*) adj3 (ontolog* or epistemolo* or pedagog* or axiolog*)).mp. |  |
| 31. 28 or 29 or 30 |  |
| 32. 7 and 18 and 27 and 31 |  |
